# Rhombencephalitis, a forgotten diagnosis in the post-partum period: a case report

**DOI:** 10.1007/s00701-025-06606-4

**Published:** 2025-07-10

**Authors:** Lejla Islamagič, Benedicte Parm Ulhøi, Gorm von Oettingen, Søren Ole Stigaard Cortnum, Anders Rosendal Korshøj, Gaston Schechtmann

**Affiliations:** 1 Department of Neurosurgery, Odense University Hospital, Odense, Denmark; 2 Department of Neurosurgery, Aarhus University Hospital, Aarhus, Denmark; 3 Department of Pathology, Aarhus University Hospital, Aarhus, Denmark

**Keywords:** Central nervous system infection, Rhombencephalitis, Post-partum infection, Immunosuppression

## Abstract

Rhombencephalitis (RE) or brainstem encephalitis is a rare but potentially deadly condition. It often affects hosts with weaker or altered immune response. A previously healthy 30-year-old female, three months post-partum, presented with a ten-day history of headache, vomiting, seizures and consciousness deterioration. MRI showed suspect brainstem glioma and hydrocephalus. Initial treatment aimed to reduce high intracranial pressure with an external ventricular drain and high-dose steroids. Despite negative blood cultures, CSF sampling was overlooked as RE was not suspected. The condition proved fatal four days later. Post-mortem histology revealed the RE as the cause of death, but PCR did not show an infectious ethology. With patients in the post-partum period with progressive neurological deterioration and FLAIR2-changes in brainstem MRI, RE should be considered early on.

## Introduction

*Rhombencephalitis (RE) is a rare, life-threatening condition characterised by inflammation of the brainstem and cerebellum. The aetiology of RE includes infections, autoimmune diseases, and paraneoplastic syndromes. In the literature, the diagnosis of RE has often been used interchangeably with brainstem encephalitis (BE), despite slight anatomical differences—BE affects only the brainstem* [9]. It more commonly affects vulnerable populations such as elderly, immunosuppressed individuals, cancer patients, and during pregnancy—it rarely affects previously immunocompetent patients [3, 5, 10]. RE is most frequently caused by a severe food-borne listeria monocytogenes bacterial infection in younger population [9]. Other common causes are infections with enterovirus 71 (primarily in the Asian-Pacific region) and herpes viruses (predominantly herpes simplex 1 (HSV1)), why it is recommended to initiate empirical treatment with antibiotics and antiviral treatment [9]. Less common causes are paraneoplastic syndromes and autoimmune diseases (the most common autoimmune ethiology is Behcet’s disease) [9, 15]. In cases of Bechet’s RE, over 90% have abnormal MRI scans and around 94% have cerebrospinal pleocytosis [9]. Other autoimmune causes of RE are multiple sclerosis, neuromyelitis optica spectrum disorder, MOG-antibodies-associated disease, connective tissue diseases and vasculitis, acute disseminated encephalomyelitis, and more [15].

The following case illustrates the diagnostic and therapeutical hurdles that can occur when RE is not suspected, and when the post-partum period is not thought to affect the immune system.

### Case

A 30-year-old previously healthy female was admitted to the emergency department with a ten-day history of severe, intermittent headache, neck pain, dizziness, vomiting and, on the day of admission, impaired consciousness. She had a history of neck pain and migraines and had three months prior to admission given birth. Two to three weeks after birth, the patient experienced neck pain, headache, dizziness, and vomiting, which was treated with iv. fluids. Two months later, the pain returned, leading to acute hospitalisation where the patient suffered a generalised tonic–clonic seizure and afterwards descended to GCS 3, necessitating intubation. A cerebral CT with angiographic sequences showed an infratentorial cystic process of 2.5 cm with aqueduct compression and severe hydrocephalus, and partial herniation of the cerebellum through foramen magnum. The angiography did not show any vascular abnormalities.

Upon arrival to the operation theatre, both pupils were dilated and non-responsive to light. There were no cornea- or ciliary reflexes. An external ventricular drain (EVD) was placed to relieve the hydrocephalus, and an intracerebral pressure probe (Raumedic, AG) was placed for monitoring the intracranial pressure (ICP). The high opening pressure upon drain placement was controlled and positive pupilar responses reappeared within minutes.

**Fig. 1:**
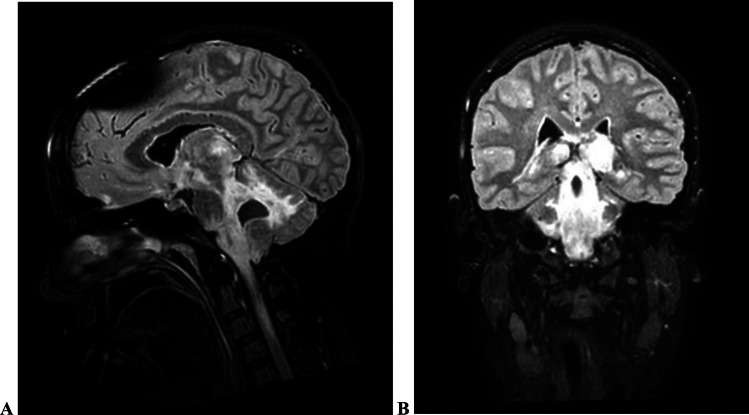
Image **A** is the sagittal T2-weighted brain MRI with FLAIR-sequence showing the extensive changes from the brainstem all the way to C2. Image **B** is the coronal diffusion weighted image showing the extent of the changes

Brain MRI showed a diffuse tumour suspect change stretching supratentorial from the left thalamus, down to the brainstem and to the cervical medulla (Fig. 1). Extensive FLAIR2-changes were observed in relation to the fourth ventricle, stretching from the left thalamus down to C2. A diffuse midline glioma CNS WHO grade 4 was suspected and high-dose steroids were initiated in consequence.

Between day 2 and 3, the ICP crept up to 30 to 40 mmHg despite sedation with midazolam 30 mg/hour, fentanyl 300 μg/hour, propofol 500 mg/hour, remifentanyl 800 μg/mL, and multiple bolus administrations of hypertonic saline. Ketamine was added at 5 mg/kg/hour, midazolam and fentanyl were increased, pCO2 was reduced. ICP decreased to around 30 mmHg with unchanged pupil size. A repeat cerebral CT-scan showed increased global cerebral swelling despite correct placement of the EVD and subsequently decreased ventricle dilatation. The ICP increased in such a degree that a thiopental-coma was added, only with a short-lived effect. No further medical or surgical options were considered available, the patient kept deteriorating despite treatment and eventually died on the fourth day.

### Laboratory examinations

The C-reactive protein (CRP) was below 4 (mg/L) upon admission but the leucocytes were elevated at 16,4 (mg/L). On Day 2, CRP increased to 91 (mg/L) and spontaneously decreased to 71 (mg/L) on Day 3. Likewise, leucocytes increased to 21.1 (10E9/L) on Day 2 and decreased to 15.3 (10E9/L) on Day 3. The patient was initially hypothermic (35.0 ֯C) and was warmed up but was sub-febrile Day 2 (38.0 ֯C) which was managed with paracetamol. The increase in CRP and leucocytes were interpreted as non-infections, thus no antibiotic treatment was initiated. Likewise, no CSF cultures were performed because infection was not suspected. Blood-cultures were negative.

### Post-mortem pathology and immunohistochemistry

**Fig. 2:**
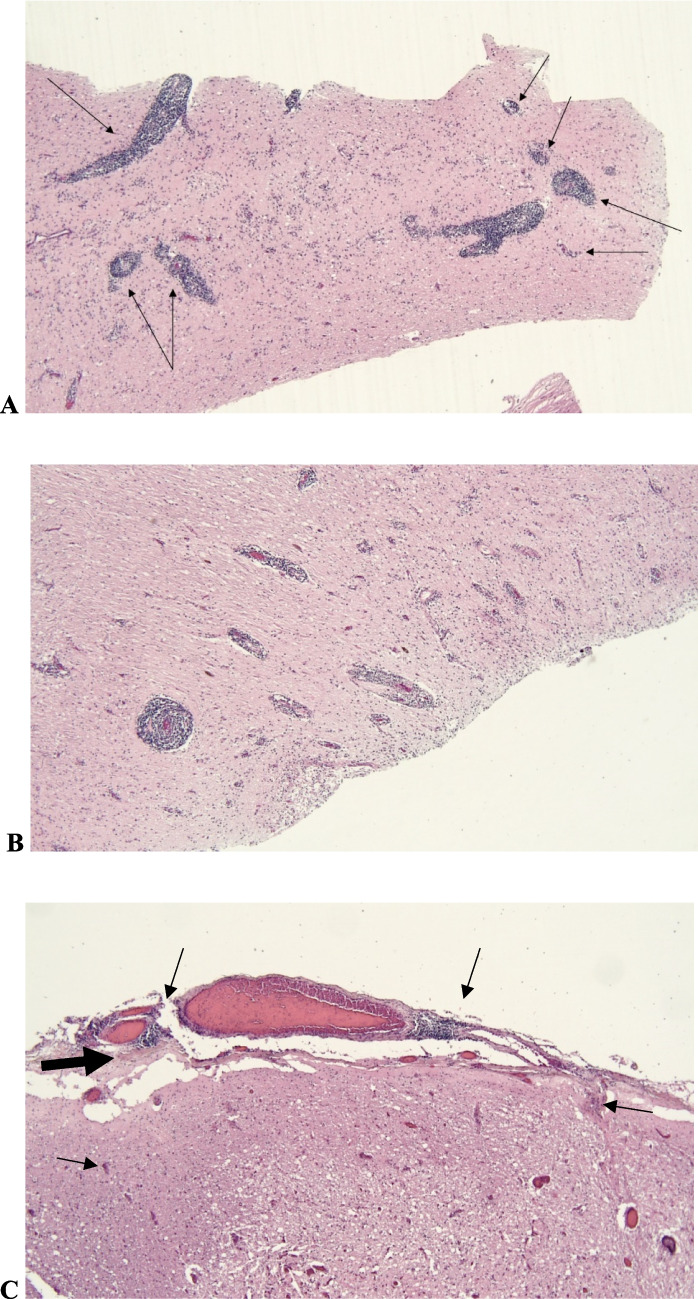
The post-mortem microscopic pathology images (40 times enhancement). **A** represent the mesencephalon and **B** represents the cerebellum, where thick sleeves of perivascular lymphocytes and a few scattered in the surrounding tissue (marked with arrows). **C** represents the meninges around pons where the two bottom short arrows point at cortex, the two top long arrows show lymphocyte infiltration in the meninges, and the thick arrow on the left shows the meningeal border. The aqueduct was compressed around 1 mm in diameter

As shown in Fig. 2, lymphocyte inflammation and microbleeds were found mostly in the parenchyma(l) and meninges of the brainstem and to a lesser degree in the cerebellum and left temporal lobe. The combination of meningeal inflammation and perivascular inflammation in the brainstem and cerebellum indicated a viral infection. Anoxic changes in the neurons of the brainstem, cerebellum, hippocampus, and cortex were also observed. Immunohistochemical analysis showed a mixture of CD3- and CD20 positive lymphocytes. The CD3 positive lymphocytes were also positive in CD4 and less positive in CD2, CD7, and CD8. No signs of tumour cells, vasculitis, lymphoma, Congophylic angiopathy, granulomas, viral inclusions, bacteria (l inclusions) or acute inflammation were found. The final diagnosis was RE.

Post-mortem immunohistochemistry and PCR was negative for HSV1, HSV2, cytomegalovirus, enterovirusis, parechovirus, bacteria, parasites and fungi.

## Discussion

This case highlights the diagnostic hurdles regarding RE due to its rarity, especially in a post-partum patient. Brain MRI with hydrocephalus and a suspected brainstem glioma, biochemical markers, and the patient’s symptoms guided clinicians towards a diagnosis of primary brain tumour and not infections or autoimmune disorders, leading to oversight of this rare but fatal diagnosis. Infection was not suspected as the patient only had leucocytosis upon admission and a normal CRP, both increased and decreased spontaneously. Furthermore, she was hypothermic upon admission and the primary brain MRI indicated an underlying malignant tumour and hydrocephalus, why high-dose steroids were administered and an EVD was inserted. Antibiotic treatment was not considered since infection was not suspected and blood cultures returned negative.

The patient was previously healthy, with no known immunodeficiencies. A consideration that was not made was the post-partum period as a potential immunocompromised state that increases the risk of infection [12]. Although the specific immunological mechanisms involved in the development of rhombencephalitis (RE) during pregnancy and the postpartum period are not yet fully understood, it is well established that a cascade of changes occurs in the immune system following childbirth. These changes are influenced by a combination of factors, including the physiological stress of delivery, hormonal fluctuations, and inflammatory responses. Singh et al. [12] describe how the rapid reversal of immunosuppressive adaptations that occur during pregnancy, to support embryonic implantation, can lead to rebound proinflammatory activity in the postpartum period. This immunological shift has been implicated in the reactivation of latent infections as well as the onset or exacerbation of autoimmune diseases.

Furthermore, it has been shown that normalisation of immune system after a pregnancy may take between 3–4 months [7], and an increase in T-cell and B-cells activity up to 10 months post-partum, which may explain autoimmune disease worsening in this period [14]. These immunological dynamics suggest that the postpartum period may represent a state of transient immune dysregulation or vulnerability, potentially increasing susceptibility to infections. In light of these considerations, we speculate that the patient’s postpartum status may have contributed to a temporary alteration of the immune system, thereby increasing her risk for developing RE during this period.

Post-partum RE is a rare condition of which few reports exist. We found few reported cases of rhombencephalitis post-partum which were associated with autoimmune disease caused by anti-centromere antibody (ACA) [8], myelin oligodendrocyte glycoprotein antibodies (MOG-IgG) [13], and extracellular labelling of live hippocampal neurons [1],of which the latter is a study in which they screened CSF for autoantibodies in ninety-six patients with post-partum psychosis. Four showed clear extracellular labelling of live hippocampal neurons, two of which had characteristic staining patterns for anti-NMDA. This prompted the authors to suggest screening these patients for CSF anti-NMDA encephalitis, especially in patients with acute psychosis and extrapyramidal symptoms.

In our case, we did not find a final ethology for the disease, despite extensive post-mortem histological examinations and PCR analysis. If RE is suspected, blood-cultures and CSF analyses are essential for diagnosis [4, 6]. A limiting factor of our report is the lack of CSF studies as it is not routinely performed after EVD-placement, and we did not suspect an infectious cause of disease why there was no clinical reason for the analysis.

It has been shown that inadequate antibiotic treatment is one prognostic factor associated with poor outcomes when RE is suspected [11]. The most common agents are listeria monocytogenes and HSV1, therefore initiation of both empiric antibiotic and antiviral treatment is recommended if the diagnosis is suspected. There is no consensus in the literature regarding the use of dexamethasone in cases with neurolisteriosis. It has been found to significantly increase the mortality in one study [2]. Oppositely, others have found no increase in mortality when using dexamethasone but a trend towards fewer neurological sequelae in the group receiving steroids [11]. Thus, there are no clear recommendations regarding the use of steroids. Treatment should therefore rely on a clinical evaluation of each patient.

In conclusion, in postpartum patients presenting with rapidly progressive neurological deficits and FLAIR- and T2-weighted changes on MRI, the diagnosis of rhombencephalitis should be considered early to improve clinical outcomes and reduce mortality. Early identification of potential causes through blood- and CSF cultures, and initiation of prompt empirical treatment are the cornerstones of patient management.

## Data Availability

No datasets were generated or analysed during the current study.
